# Identifying rare-variant associations in parent-child trios using a Gaussian support vector machine

**DOI:** 10.1186/1753-6561-8-S1-S98

**Published:** 2014-06-17

**Authors:** Ake T Lu, Rita M Cantor

**Affiliations:** 1 Department of Human Genetics, David Geffen School of Medicine, University of California at Los Angeles, Los Angeles, CA 90095, USA; 2 Center for Neurobehavioral Genetics, David Geffen School of Medicine, University of California at Los Angeles, Los Angeles, CA 90095, USA

## Abstract

As the availability of cost-effective high-throughput sequencing technology increases, genetic research is beginning to focus on identifying the contributions of rare variants (RVs) to complex traits. Using RVs to detect associated genes requires statistical approaches that mitigate the lack of power with the analysis of single RVs. Here we report the development and application of an approach that aggregates and evaluates the transmissions of RVs in parent-child trios. An initial score that incorporates the distortion in transmission of the observed RVs from the parents to their offspring is calculated for each variant. The scores are analyzed using a support vector machine that handles these data by mapping the transmission distortion of the multiple RVs into a one-dimensional score in a nonlinear fashion when parent-child trios with affected and nonaffected children are contrasted. We refer to this approach as Trio-SVM. A total of 275 trios were available in the Genetic Analysis Workshop 18 data for analysis. Because of their nonindependence and the extended linkage disequilibrium (LD) within pedigrees, Trio-SVM was vulnerable to type I errors in detecting association. Using the GAW18 data with simulated trait values, Trio-SVM has an appropriate type I error, but it lacks power with a sample of 267 trios. Larger samples of 500 to 1000 trios, derived from combining the simulated data, provided sufficient power. Two chromosome 3 candidate genes were tested in the real GAW18 data with Trio-SVM, and they showed marginal associations with hypertension.

## Background

Genome-wide association studies (GWAS) of common variants have not explained the heritability estimates of common complex disorders [1]. In response, exome sequencing, which is designed to reveal rare variants (RVs) with a frequency less than a value in the range of 1% to 5%, is being applied to pursue additional risk genes. Interpretation of RVs is best done for Mendelian disorders within pedigrees to identify significant loci and avoid artifacts of the sequencing process. However, for complex disorders and quantitative traits, RVs that segregate only within a few pedigrees do not provide adequate statistical power to implicate a particular gene when they are analyzed alone. Approaches to solve this problem involve the aggregation of RVs within genes and regions. We developed an approach, called Trio-SVM, using the support vector machine (SVM) method that aggregates and tests the RVs of a gene for a dichotomized trait in parent-child trios [2]. Parent-child trios are used to test association through distortions in transmission from the parents to their children. An advantage of this approach is that (a) the transmission of RVs can be aggregated across genes and compared with their aggregation in controls by the SVM, and (b) population stratification is mitigated because only parents with the RV provide information in the analysis. That is, the differences in frequencies of RVs in different ethnic groups will have no effect on the test statistic because only opportunities for transmission in parents heterozygous for RVs contribute to the transmission distortion data used by the SVM.

Using Trio-SVM, all members of the trios are sequenced for RVs, and the observed RV transmissions are compared with what is expected, given the parental RV genotypes. Transmission distortions in a gene are combined using SVM. The area under receiver operating characteristic (area under the curve [AUC]) was generated by SVM when contrasting the transmission between affected and unaffected children is estimated for each gene under analysis and used as the test statistic. The strength of Trio-SVM is that it allows for each RV to either confer risk or to be protective and contribute to an overall score in which the direction of the effect for each RV is not a factor in the score.

One potential concern is the availability and choice of control groups for the SVM. First, the control sample should be beyond the age of risk for the disorder under analysis and have appropriate environmental exposures when those are known to be important. Second, to provide an opportunity for transmission of RVs from parents to their children, ethnic matching, although not necessary, may be helpful. An interesting choice might be the unaffected siblings from the trios in the study because they would have the same opportunities to inherit the RVs that are transmitted to their affected siblings.

## Methods

### An overview of support vector machine

The purpose of the SVM is to discriminate between two groups using a set of variables. It is particularly useful when the number of variables is greater than the number of individuals in the data set. SVM is based on a model with N ordered pairs $\left(y_{i},x_{i}\right)$ where $y_{i}$ is a binary outcome with a vertex −1 assigned to one group and +1 to the other and, $x_{i}=\left(x_{ij}\right),j=1,2,..,M$, is a vector with *M *predictors.

If "." denotes the dot product and "^" the parameter estimate, SVM constructs two hyperplanes in space, $H_{1}:x_{i}.w+b=-1$ and $H_{2}:x_{i}.w+b=+1$ in which the weights *w *and the offset *b *are estimated to maximize the separation of $\left(\frac{2}{||w||}\right)$between $H_{1}$ and $H_{2}$, with the constraint $y_{i}\left(x_{i}.w+b\right)-1\geq 0;\forall i$ (i.e., all of the observations of two groups are separated by the two hyperplanes). The optimization is equivalent to minimizing $L_{p}=\frac{1}{2}{∥w∥}^{2}+\overset{N}{\underset{i}{\sum }}\alpha_{i}\left(1-y_{i}\left(x_{i}.w+b\right)\right)$, with respect to *w *and *b*, where $\alpha_{i}\geq 0$ are Lagrange multipliers. Geometrically, $\hat{w}$ is a function of $N_{s}$ support vectors, with nonzero $\alpha_{i}$ that locate on the margins of $H_{1}$ and $H_{2},$and the solution of $w\;.x_{i}$ is given by $\overset{N_{s}}{\underset{s}{\sum }}\alpha_{s}y_{s}x_{s}.\;x_{i}$.

SVM provides the advantage of allowing Mtobe>N because the solution that estimates *w *is based on the support vectors. An additional advantage is the relaxation of linear mapping by using a kernel function *K *that corresponds to a nonlinear function *φ *such that $x_{i}$ is replaced by $\varphi \left(x_{i}\right)$ and $x_{s}.x_{i}$ is replaced by $\left(x_{s},x_{i}\right)=\varphi \left(x_{s}\right).\varphi \left(x_{i}\right)$. Using a Gaussian kernel with a scale, $\sigma_{g}^{2}$, the dot product $\hat{w}\;.\varphi \left(x_{i}\right)$ is expressed as $\overset{N_{s}}{\underset{s}{\sum }}\alpha_{s}y_{s}\text{exp}\left(-{∥x_{s}-x_{i}∥}^{2}/2\sigma_{g}^{2}\right)$. A penalty term (in general denoted by *C*) is added for a generalization of the optimal hyperplane when the data do not allow the two groups to be completely separated, which limits the Lagrange multipliers to range between 0 and C.

### Adapting support vector machine for parent-child trios: Trio-SVM

Trio-SVM analyzes a set of N parent-child trios, in which each child is described by a coordinate $\left(y_{i},x_{i}\right)$, for the M RVs observed for that child and his or her parents and $y_{i}$ is -1 when the child is affected and +1 otherwise. Here $x_{i}$ incorporates the conditional distribution of RVs on parental genotypes using the framework of the family-based association test (FBAT) [3]. At each RV site j, $x_{ij}$ is the difference between the observed and expected transmission of an RV to the child given the two parental genotypes for that RV. Using this, each child then gets a composite score for the test gene, $y_{i,score}$, which is modeled by $\hat{w}\;.\varphi \left(x_{i}\right)+\hat{b}$, which aggregates the RV. The AUC (denoted by θ) of $y_{score}$ for H_0_: θ <= 0.5 vs H_a_: θ > 0.5 is used to represent the composite scores for distorted transmission within a gene over the sample of trios comparing those who are affected with those who are not. Accepting that θ is greater than 0.5 indicates the combined RVs in the gene are transmitted with greater distortion from that which is expected in the cases when compared with the control participants. The test is one-sided because each group is assigned to a fixed vertex; the statistic $\hat{\theta }/SE\left(\hat{\theta }\right)$ is asymptotically Gaussian. For case-control analyses, one would let $x_{ij}$ count the number of RVs at each site.

### Applying Trio-SVM to Genetic Analysis Workshop 18 pedigree data

For the GAW18 data, Trio-SVM was used to combine all observed RVs for a given gene, by selecting all affected and unaffected individuals having both parents in each pedigree and treating them as independent. A total of 275 such trios were derived from the 959 individuals in 20 GAW18 pedigrees ascertained for type 2 diabetes (T2D). The pedigree members were genotyped at 472,049 SNPs on GWAS platforms. Half of the sample (*n *= 464) was sequenced at 8,348,674 sites, and imputation of nonsequenced individuals was performed using the GWAS data, thus providing each individual with a constellation of RVs. Blood pressures were taken longitudinally at from 1 to 4 exams for 932 participants. Hypertension was assigned based on systolic blood pressure (SBP) greater than 140 mm Hg, diastolic blood pressure (DBP) greater than 90 mm Hg, or use of antihypertensive medications.

Trio-SVM accepted the input of the GAW18 pedigree data in linkage format, and the noninformative sites in which no RVs were observed were removed. All trios with two parents available were gleaned from the pedigrees and treated as if they were independent for these analyses. However, because they are not independent and LD reaches much greater distances in pedigrees than in independent samples, significant results with Trio-SVM may lead to false positives in such pedigrees. Specifically, for a disorder, if there is a causal common variant, its haplotype will segregate with the disorder throughout the pedigree. Any RVs that are on the haplotype in the pedigrees will be carried along with it, and genes that happen to have many RVs on that haplotype will be implicated. If the RVs are not in the causal gene, a type I error regarding association will occur.

Analyses were focused on chromosome 3, as suggested by the GAW18 organizers. Two T2D GWAS candidate genes, *ADCY5 *at (3q21.1) [4] and *UBE2E2 *(3p24.2) [5], on a different arm of chromosome 3 were tested using Trio-SVM. For comparison, SVM without transmission information was used to analyze 108 founders consisting of 67 cases and 41 control participants.

### Trio-SVM type I and type II error rates using the simulated pedigree data

Two hundred replicates of the genotyped data in the GAW18 pedigrees with the trait simulated under specific genetic models were available for assessments of type I and II statistical errors. The genes on chromosome 3 that were predisposing and nonpredisposing in the simulated models were tested. RVs were included in the analysis when their frequencies were less than 0.01 and less than 0.03 in two separate assessments. These analyses were performed on the simulated trait, hypertension, defined in two ways: (a) adjusted by age, age × gender, gender, and use of antihypertensive medications and (b) not adjusted. Covariates were included using linear mixed models with a random effect to account for the intrapedigree correlation. The traits were adjusted to age 38, no medications, and male gender. Power analyses were based on evaluating the predisposing gene *MAP4*, and the type I errors were assessed for the nonpredisposing gene, *ARL13B *(93.8 Mb), located between *RYBP *(72.5Mb) and *B4GALT4 *(118.9Mb), where both influenced DBP or SBP. To evaluate the power in a larger sample size, 500 and 1000 trios were drawn from the 200 replicates using bootstrap sampling.

Trio-SVM was evaluated using a Gaussian kernel ($\sigma_{G}^{2}$ fixed at 1) and 5-fold cross-validation for model selection across different C, from 1 to 10.

## Results and discussion

### Trio-SVM analysis of type 2 diabetes candidate genes

Table 1 reports the results of the two candidate genes tested for association in the GAW18 pedigrees with Trio-SVM. They both show association with hypertension with *p*-values of 3.2E-04 for *ADCY5 *at 3q21.1 and 0.035 for *UBE2E2 *at 3p24.2. For this analysis, genes were selected on both arms of chromosome 3 because we wanted to see if we could detect independent signals given the poor resolution because of LD in pedigrees. Association in the set of 108 founders, in which 58 independent cases were compared with 50 independent control participants, was not detected. However, this limited sample provides very low power. A significant *p*-value for the AUC statistic has two possible interpretations. Either a consistent set of SNPs is responsible for the signals in a gene or different variants are contributing to the signals in the different pedigrees [6]. However, disentangling these with the large number of variants contributing to these signals is not straightforward.

**Table 1:** Trio-SVM analyses of candidate genes in GAW18 trios and founders

		Trios (*n *= 275) 66 case trios 209 control trios		Founders (*n *= 108) 50 cases founders 58 control founders	
**Gene (#Bp)**	**#RV sites**	**AUC (SE)**	***p*-Value**	**AUC (SE)**	***p*-Value**

*ADCY5 *(166,249)	426	0.637 (0.040)	3.2E-04	0.554 (0.056)	0.17
*UBE2E2 *(387,512)	917	0.575 (0.041)	0.035	0.539 (0.057)	0.25

*AUC*, the area under the curve; *RV*, rare variant; *SE*, standard error.

### Trio-SVM: power and type I error

Table 2 summarizes the assessments of power and type I error. In the adjustment, we used the averages of slope estimates over 200 linear mixed models on the 200 replicates. The powers were increased by the adjustment. Of the sites with minor allele frequencies (MAFs) less than 0.03, 9 individual variants explained a total variance of 1.73 to 2.04% in DBP or SBP traits while the variant, temp_323826, was removed in the set of MAF less than 0.01 that explained a total variance of 0.8 to 0.9%. The maximum power, 0.19, was achieved by using the adjusted simulated traits and was increased by 0.05 as adding 91 sites (0.01 <MAF <0.03). The small increment may be a result of the fact that the sites in LD with the functional variant, temp_323826, were already included in the group with MAFs less than 0.01. Subsequently, we evaluated the power of MAFs less than 0.03 using large samples generated from the bootstrap and the adjusted simulated traits (Table 3). A 0.80 power at 0.05 α was nearly reached using 500 trios, and the power was over 0.80 at a more stringent α (0.0001) using 1000 trios. It is of importance that type I errors distributed around 0.05 and were not inflated by the adjustment.

**Table 2:** Trio-SVM type I error and power in GAW18 simulated data (267 trios)

RV frequency	Trait adjusted	#RV sites	Power	#RV sites	Type I error (for *p-*values <0.05)
<0.03	No	405	0.15	115	0.040
	Yes		0.19		0.055
<0.01	No	314	0.11	91	0.065
	Yes		0.14		0.065

*RV*, rare variant.

**Table 3:** Trio-SVM power in multiple replicates

α^1^	500 trios	1000 trios
*p-*Value <0.05	0.755	0.995
*p-*Value <0.01	0.610	0.980
*p-*Value <0.001	0.405	0.930
*p-*Value <0.0001	0.215	0.870

^1^Level of significance for the gene under analysis.

## Conclusions

Applications of machine learning methods in genomic data are just beginning [7-9]. Using SVM, we developed a novel approach for analysis of RVs to handle high-dimensional genomic data, relax a linear relationship between $\left(y_{i},x_{i}\right)$, and control population stratification. One disadvantage is that the magnitude of $\hat{w}$ cannot be explicitly expressed by using a nonlinear kernel. Importantly, we can detect the association between RVs and a test trait when applying Trio-SVM to a sample composed of nuclear families. Our future work is to increase the power by considering other newly defined kernel functions, such as wavelet transform, and make the extension a viable option in our code. The MATLAB code of Trio-SVM can be obtained from the authors.

## Competing interests

The authors declare that they have no competing interests.

## Authors' contributions

ATL and RMC designed the overall study and drafted the manuscript. ATL wrote the code of Trio-SVM and conducted statistical analyses. RMC coordinated the conception of the study and wrote the final draft of the manuscript. All authors read and approved the final manuscript.
